# The role of subjective cognitive complaints in self-management among haemodialysis patients: a cross-sectional study

**DOI:** 10.1186/s12882-022-02994-2

**Published:** 2022-11-14

**Authors:** Frederick H. F. Chan, Stanton Newman, Behram A. Khan, Konstadina Griva

**Affiliations:** 1 Population/Global Health, Lee Kong Chian School of Medicine, Nanyang Technological University, Singapore, Singapore; 2 School of Health Sciences, Division of Health Services Research and Management, City University of London, London, UK; 3 National Kidney Foundation, Singapore, Singapore; 4 Yong Loo Lin School of Medicine, National University of Singapore, Singapore, Singapore

**Keywords:** End-stage renal disease, Haemodialysis, Cognitive impairments, Subjective cognitive complaints, Self-efficacy, Treatment adherence, Self-management

## Abstract

**Background:**

Subjective cognitive complaints refer to self-experienced difficulties with everyday cognitive tasks. Although there has been a fair amount of research on cognitive impairments and cognitive complaints in end-stage renal disease, the practical implications of these complaints remain unclear. The current study aims to examine the associations of cognitive complaints with sociodemographic and clinical variables, mood, as well as key patient-reported outcomes, i.e., self-efficacy, self-management skills, and treatment adherence.

**Methods:**

A total of 305 haemodialysis patients (mean age = 53.97 years, 42.6% female) completed the Kidney Disease Quality of Life Cognitive Function subscale, a brief measure of cognitive complaints. The recommended cut-off point of 60 was used to identify probable cognitive impairment. Measures of self-efficacy, self-management skills (i.e., symptom coping, health monitoring, health service navigation), treatment adherence, and mood symptoms were also administered. Between-group comparisons and correlational analyses were performed to examine associations of cognitive complaints with sociodemographic, clinical, and health behaviour variables. Mediation analyses were also conducted to investigate the mediating role of self-efficacy on the relationship between cognitive complaints and treatment adherence.

**Results:**

Nearly a quarter (23.0%) of haemodialysis patients reported cognitive complaints indicative of clinical impairments. Risk of probable impairments was higher for patients with hypertension, diabetes, those diagnosed with end-stage renal disease at an older age, and those with shorter time on dialysis. Subjective cognitive complaints (both rates of probable impairments as per cut-off and continuous scores) were significantly associated with lower disease and treatment self-efficacy, poorer self-management skills, lower treatment adherence, as well as higher symptoms of distress. Mediation analysis indicated that treatment self-efficacy mediated the relationship between cognitive complaints and treatment adherence.

**Conclusions:**

The current study demonstrated the clinical characteristics of haemodialysis patients who report cognitive complaints indicative of probable cognitive impairments and showed the associations of these complaints with self-management outcomes. Future studies should adopt more comprehensive measures of cognitive complaints and longitudinal designs to confirm the current findings.

## Background

The burden of cognitive impairments (CIs) in end-stage renal disease (ESRD) has been extensively studied in the past decades. Cognitive dysfunction starts to manifest in early renal dysfunction [1, 2], persists along the course of kidney disease progression, and is not fully reversible by dialysis or transplantation [3–6]. Wide ranging deficits in domains such as attention, memory, and executive function are frequently seen in haemodialysis (HD) patients and the prevalence of CIs is higher in HD patients compared to the general population [4, 7]. CIs in HD patients are associated with increased risks of functional disability, hospitalisation, mortality, and dialysis withdrawal [8–10]. These effects are thought to be due to CIs interfering with self-management capabilities, adherence behaviours, and decision-making processes [2, 11, 12], however studies directly testing these associations are lacking.

To date, the practical implications of CIs in ESRD patients on HD treatment are not well understood. Most prior work focused on objective cognitive function assessed by neuropsychological tests [2, 3, 11, 13], which albeit sensitive in detecting CIs, may have limited relevance to patients’ experiences of difficulties in everyday cognitive activities [14–16]. Some researchers have questioned the extent to which performance on these neuropsychological tests reflect patients’ cognitive performance in real-world settings. Indeed, one study found that it was dialysis patients’ self-reported cognitive function, but not objective cognitive function, that was associated with daily functioning assessed by the basic and instrumental Activities of Daily Living scales [14]. This finding suggests that subjective cognitive complaints (SCCs), the self-experienced difficulties in performing everyday cognitive tasks, may have additional utility in predicting real-world patient outcomes, and may improve the real-world meaningfulness of objective cognitive assessments.

Currently, we have very limited understanding of the association between SCCs and important patient-reported outcomes in HD patients such as self-management skills and treatment adherence. Self-management of ESRD requires various skills including monitoring of health condition (e.g., weight gain), coping with symptoms, navigating healthcare services, communicating with the healthcare team, as well as problem-solving and decision-making for the treatment [17]. HD patients are also required to adhere to complex guidelines concerning their diet, fluid intake, and medication. These processes rely on patients’ cognitive function because they must be able to understand and remember their medical regimen, and to develop and implement a plan to adhere to their regimen [18]. It is therefore plausible that HD patients who report more frequent and severe SCCs may have worse self-management skills and treatment adherence. Poor self-management may then result in increased hospitalisation, mortality, and healthcare costs [19]. It is also plausible that SCCs may influence behavioural outcomes through an indirect pathway by reducing patients’ self-efficacy. Self-efficacy refers to an individual’s perceived capability or confidence to perform a target behaviour [20]. Greater self-efficacy has been shown to be associated with better treatment adherence as well as better clinical outcomes (e.g., lower serum potassium and inter-dialytic weight gain) in ESRD patients [19]. Less well understood are the associations of SCCs with self-efficacy and adherence behaviours. In the context of ESRD, there are high cognitive demands related to disease self-management and adherence to the complex medical guidelines. The self-awareness of cognitive deficits (i.e., SCCs) may compromise patients’ self-confidence and motivation towards self-management, thereby adversely impacting adherence behaviours.

An additional research gap evident in the literature is that most previous studies assessed SCCs as a continuous variable. However, the Kidney Disease Quality of Life Cognitive Function subscale (KDQOL-CF) has a validated cut-off score that is able to differentiate dialysis patients with and without probable CIs [21]. Only one study reported the prevalence of probable CIs detected by this cut-off score [22], and no study has used this cut-off to explore the sociodemographic or clinical characteristics of ESRD patients with probable CIs, or the utility of this cut-off in predicting important patient outcomes such as adherence.

To address these gaps and advance current understandings, this study aimed: (1) to document the prevalence of probable CIs as indicated by KDQOL-CF in HD patients, (2) to identify risk profiles for probable CIs based on sociodemographic and clinical variables, (3) to examine the predictive ability of KDQOL-CF scores (both continuous and categorical) for key patient-reported outcomes in HD, i.e., self-efficacy, self-management skills, treatment adherence, and mood symptoms, and (4) to explore the potential mediating effects of self-efficacy on the relationship between SCCs and treatment adherence.

## Methods

### Design

The current study is based on data from two studies undertaken at the same period in National Kidney Foundation (NKF) of Singapore: an observational study of incident HD patients [23] and baseline (i.e. pre-randomisation) data from a randomised controlled trial evaluating the effectiveness of a self-management intervention for HD patients [24]. NKF is a non-profit organisation in Singapore that provides community-based HD treatment. The study protocols were approved by the institutional review board of National University of Singapore and are in compliance with the Helsinki declaration [25]. All study participants provided written informed consent prior to study enrolment and assessments. Details of the two original studies can be found in previous publications [23–26].

### Participants

Participants from both studies were recruited from 14 out of 24 NKF dialysis centres across Singapore through convenience sampling. These 14 centres were purposefully selected to ensure geographical representation of dialysis centres island-wide; the remaining were not included due to the lack of facilities to host the intervention for the randomised controlled trial or distance from other dialysis centres with such facilities [25]. Although it would have been preferable to recruit patients from all 24 centres during the study period, this was not deemed feasible. Nevertheless, no difference in sociodemographic or ethnic composition of patients across centres was expected due to implementation of hosing policies in Singapore to ensure adequate representation of ethnic groups in public housing projects [27].

Participants were recruited subject to following inclusion criteria: 21 years of age or older, established on HD treatment in one of the 14 NKF dialysis centres, and fluent in either English, Mandarin or Malay. Individuals were excluded if they had documented diagnosis of major sensory, motor, or cognitive impairments that would prohibit informed consent. Patients who were only fluent in dialects or Tamil were excluded because there were no resources in the study team allowing for delivery of intervention or administration of questionnaires in these languages. Patients with limited life expectancy due to comorbidity such as advanced stage/terminal malignancy were also excluded because the original randomised controlled trial included multiple intervention sessions and assessments which were considered too demanding for these patients. The eligibility criteria were identical between the two original studies.

### Procedure

A list of eligible patients was provided by the nurse manager in each participating dialysis centre who were aware of the eligibility criteria. A research team member fluent in the patients’ preferred language approached each eligible patient for invitation to the studies. Patients were administered the questionnaires if they consented to participate. The questionnaires administered were identical between the two original studies. Upon completion, participants were given a small cash reimbursement.

### Measures

#### Sociodemographic and clinical information

Self-reported demographic information was collected from each participant including gender, age, ethnicity, educational level, marital and employment status, and household income. Clinical variables extracted from the medical record included age at ESRD diagnosis; primary kidney disease diagnosis; time on HD; presence of cerebrovascular disease, hypertension, and diabetes; dialysis adequacy (Kt/V); biochemical lab assays, i.e., serum phosphorus and serum potassium; and relative interdialytic weight gain (IDWGr), which is the ratio of absolute IDWG to a patient’s dry weight at each midweek dialysis session during the assessment period.

The Charlson Comorbidity Index (CCI) was used to consolidate patients’ comorbidity burden [28, 29]. The nurse manager of each participating dialysis centre rated the CCI for patients receiving treatment in the corresponding centre. CCI scores were computed based on the methods described by Beddhu et al. [30].

#### Subjective cognitive complaints

SCCs were measured by the Kidney Disease Quality of Life Cognitive Function subscale (KDQOL-CF). The KDQOL is a measure of quality of life in patients with kidney disease [31, 32] and has been validated in Singaporean dialysis patients [33, 34]. The KDQOL-CF is one of the subscales and measures SCCs using three brief items: “During the past 4 weeks, how much of the time did you (1) react slowly to things that were said or done, (2) have difficulty concentrating or thinking, and (3) become confused” [21]. Participants were asked to respond on a six-point Likert scale ranging from “none of the time” to “all of the time” [21]. The total score of KDQOL-CF ranges from 0 to 100, with higher scores indicating better self-perceived cognitive functioning [31, 32]. A cut-off point of 60 on the KDQOL-CF has been derived by Kurella and colleagues to differentiate ESRD patients with and without probable CIs [21]. Cronbach’s alpha for the KDQOL-CF was 0.81.

#### Self-efficacy

Disease and treatment self-efficacy were measured using two scales: the 6-item Self-Efficacy to Manage Chronic Disease scale to measure self-efficacy for general demands of chronic disease (e.g., “How confident are you that you can keep the physical discomfort or pain of your disease from interfering with the things you want to do”) [35] and a dialysis-specific scale, the Self-Efficacy to Adhere to Treatment Recommendations scale. The latter was developed following formative qualitative work with HD patients [36], review by expert panel of renal health professionals, and a pilot with four HD patients [25]. This treatment self-efficacy scale contains eight items that assess participants’ self-confidence to adhere to their treatment recommendations related to fluid intake, diet, and medication (e.g., “How confident are you that you can limit your fluid intake”). For both questionnaires, participants were asked to rate on a 10-point Likert scale, ranging from “not at all confident” to “totally confident” [35]. Higher total scores indicate higher disease or treatment self-efficacy. Cronbach’s alpha was 0.92 for the disease self-efficacy scale and 0.91 for treatment self-efficacy scale.

#### Self-management skills

Self-management skills were measured by the skills and technique acquisition, self-monitoring and insight, and health services navigation subscales of the Health Education Impact Questionnaire [37]. The skills and technique acquisition subscale assesses knowledge-based skills and techniques that help patients manage symptoms or health problems (e.g., “When I have symptoms, I have the skills that help me cope”) [37]. The self-monitoring and insight subscale assesses patients’ ability to monitor their health condition (e.g., “I carefully watch my health and do what is necessary to keep as healthy as possible”) [37]. The health services navigation subscale measures patients’ ability to communicate and negotiate with healthcare providers to get their needs met (e.g., “I communicate very confidently with my doctor about my healthcare needs”) [37]. Higher scores indicate better self-management skills in these domains. Cronbach’s alpha was 0.66 for skills and technique acquisition subscale, 0.76 for self-monitoring and insight subscale, and 0.80 for health services navigation subscale.

#### Treatment adherence

Medication adherence was assessed with the Medication Adherence Report Scale (©Professor Rob Horne) [38–40] that includes 5 items (e.g., “I alter the dose”) rated on a five-point Likert scale ranging from “never” to “always. Higher total scores signify higher medication adherence. Cronbach’s alpha for this questionnaire was 0.75. To measure adherence with regards to the other treatment aspects, we used the 25-item Renal Adherence Behaviour Questionnaire that comprises 5 subscales: fluid restrictions, potassium and phosphate intake, sodium intake, adherence in times of particular difficulty, and self-care, all rated on a five-point scale ranging from “never” to “always” [41]. Higher subscale and total scores indicate higher adherence. Cronbach’s alpha for this questionnaire was 0.83.

#### Mood symptoms

Given the consistently documented associations between SCCs and mood [42–45], the Hospital Anxiety and Depression Scale was used [46, 47]. This questionnaire is a 14-item self-report measure that assesses depression (7 items; e.g., “I feel miserable and sad”) and anxiety (7 items; e.g., “I get sudden feelings of panic”). Individuals respond to each item on a four-point Likert-type scale that varies depending on the item, but generally reflects frequency (e.g., 0 = not at all to 3 = all the time) during the past week. Higher scores indicate more severe depressive or anxious symptoms. Cronbach’s alpha was 0.71 for the depression subscale and 0.82 for the anxiety subscale.

### Statistical analyses

Descriptive statistics were computed for demographic, clinical, and patient-reported variables. Two variables were derived from the KDQOL-CF scores: (1) binary classification of KDQOL-CF scores into “probable CIs” vs. “no CI” groups based on the validated cut-off [21]; (2) total sum score of the KDQOL-CF as a continuous variable to indicate frequency of SCCs in line with prior work [48]. To examine associations between SCCs and patients’ sociodemographic and clinical profile, independent samples t-tests and Chi-squared analyses were performed to examine differences between the “probable CIs” and “no CI” groups. We also performed Chi-squared analysis and ANOVA to examine difference in rates of probable CIs and continuous SCC scores between patients on HD ≤ 6 months (i.e., newly initiated HD); patients between 7–24 months on HD; and patients on HD > 24 months. To examine associations between SCCs and patient-reported outcomes, independent samples t-tests were then performed to examine differences between the “probable CIs” and “no CI” groups in their self-efficacy, self-management skills, treatment adherence, and mood symptoms. This set of analyses were then repeated using the continuous KDQOL-CF scores to determine linear associations between SCCs and patient-reported outcomes. Finally, we tested the mediating effect of treatment self-efficacy on the relationship between SCCs and treatment adherence using the PROCESS Macro [49]. Specifically, bias-corrected bootstrapped mediation analyses with 5000 bootstrapped resamples were performed to examine whether there was an indirect effect of the continuous KDQOL-CF scores on medication adherence and adherence to other treatment aspects through treatment self-efficacy. A statistically significant association or difference was considered to be present when a two-tailed *p* value was equal to or less than 0.05. Two-tailed tests were used because no specific direction was hypothesised for the associations between SCCs and self-management outcomes due to limited existing data supporting these associations.

## Results

### Assumptions

There was no evidence of violation of assumptions of normality in the current dataset. Skewness and kurtosis of all main measures (i.e., KDQOL-CF, self-efficacy, self-management skills, treatment adherence, and mood symptoms) were examined and results fell within the acceptable ranges (i.e., skewness within ± 2 and kurtosis within ± 7) suggested by previous literature [50, 51]. For independent samples t-tests, Levene’s tests for equality of variances were performed. If significant inequality in variances was observed, the t-test statistics were adjusted and reported based on modified degrees of freedom. Regarding correlation and mediation analyses, scatterplots were scrutinised to ensure that the associations are best described with linear relations.

### Sample characteristics

Of the 1076 patients screened, 652 were eligible for the study and 305 (response rate = 46.8%) provided consent and completed baseline assessments. As seen in Table 1, participants had an average age of 53.97 (SD = 11.06). The majority of patients were male (57.4%), of Chinese (54.5%) or Malay (37.7%) ancestry, received secondary education or lower (84.0%), were in a relationship (67.2%), unemployed (58.9%), and reported a household income lower than S$2000 a month (61.4%). The mean time on HD was 55.73 months. The majority of patients were on HD for more than two years (60.3%) while 22.6% initiated HD in the past six months. The average KDQOL-CF score was 70.40 (SD = 22.18) which indicated that patients experienced SCCs from “a little of the time” to “some of the time” on average. Seventy patients (23.0%) scored below the threshold of 60 on KDQOL-CF, indicating probable clinical CIs [21].

**Table 1:** Sociodemographic and clinical characteristics of study participants

	Total (*N* = 305)	Probable CIs (*N* = 70)	No CI (*N* = 235)	*p* value	Effect size
	Mean (SD) / N (%)				
Sociodemographic					
Gender				.435	0.05
Female	130 (42.6%)	27 (38.6%)	103 (43.8%)		
Male	175 (57.4%)	43 (61.4%)	132 (56.2%)		
Age (years)	53.97 (11.06)	55.70 (10.05)	53.45 (11.31)	.136	0.20
Ethnicity				.268	0.12
Chinese	162 (54.5%)	45 (64.3%)	117 (51.5%)		
Malay	112 (37.7%)	20 (28.6%)	92 (40.5%)		
Indian	22 (7.4%)	5 (7.1%)	17 (7.5%)		
Others	1 (0.3%)	0 (0.0%)	1 (0.4%)		
Highest Education				.160	0.08
Secondary or lower	252 (84.0%)	60 (89.6%)	192 (82.4%)		
Post-secondary or higher	48 (16.0%)	7 (10.4%)	41 (17.6%)		
Relationship status				.615	0.03
In a relationship	201 (67.2%)	44 (64.7%)	157 (68.0%)		
Not in a relationship	98 (32.8%)	24 (35.3%)	74 (32.0%)		
Working status				.798	0.02
Working	108 (41.1%)	23 (42.6%)	85 (40.7%)		
Not working	155 (58.9%)	31 (57.4%)	124 (59.3%)		
Household income				.906	0.01
S$2000 or below	148 (61.4%)	36 (62.1%)	112 (61.2%)		
Above S$2000	93 (38.6%)	22 (37.9%)	71 (38.8%)		
Clinical					
Age at ESRD diagnosis	45.67 (14.28)	48.73 (14.39)	44.79 (14.15)	.046*	0.28
Primary kidney disease diagnosis				.135	0.18
Diabetic nephropathy	107 (39.2%)	31 (51.7%)	76 (35.7%)		
Primary glomerulonephritis	65 (23.8%)	10 (16.7%)	55 (25.8%)		
Hypertension	20 (7.3%)	2 (3.3%)	18 (8.5%)		
IgA nephropathy	23 (8.4%)	7 (11.7%)	16 (7.5%)		
Polycystic kidney disease	8 (2.9%)	2 (3.3%)	6 (2.8%)		
Others/uncertain aetiology	50 (18.3%)	8 (13.3%)	42 (19.7%)		
Time on HD (months)	55.73 (57.70)	38.93 (44.08)	60.79 (60.39)	.001**	0.38
0–6 months	67 (22.6%)	22 (32.4%)	45 (19.7%)	.008**	0.18
7–24 months	51 (17.2%)	16 (23.5%)	35 (15.3%)		
Beyond 24 months	179 (60.3%)	30 (44.1%)	149 (65.1%)		
Presence of cerebrovascular disease				.182	0.08
No	265 (90.4%)	56 (86.2%)	209 (91.7%)		
Yes	28 (9.6%)	9 (13.8%)	19 (8.3%)		
Presence of hypertension				.027*	0.13
No	24 (8.2%)	1 (1.5%)	23 (10.1%)		
Yes	269 (91.8%)	64 (98.5%)	205 (89.9%)		
Presence of diabetes				.040*	0.12
No	154 (52.7%)	27 (41.5%)	127 (55.9%)		
Yes	138 (47.3%)	38 (58.5%)	100 (44.1%)		
CCI	4.96 (2.15)	5.34 (1.92)	4.85 (2.20)	.107	0.23
Kt/V	1.47 (0.48)	1.39 (0.29)	1.49 (0.52)	.124	0.22
Serum phosphorus	5.31 (3.44)	4.89 (1.35)	5.42 (3.82)	.274	0.16
Serum potassium	4.74 (0.69)	4.63 (0.77)	4.77 (0.77)	.151	0.21
IDWGr	3.85 (1.08)	3.80 (1.09)	3.86 (1.07)	.697	0.06

Effect sizes are either Cohen's d (t-test) or Carmer's V (Chi-squared). *CI* Cognitive impairments, *SD* Standard deviation, *N* Sample size, *ESRD* End-stage renal disease, *HD* Haemodialysis, *CCI* Charlson comorbidity index, *Kt/V* Dialysis adequacy, *IDWGr* Relative interdialytic weight gain

* *p* < .050

** *p* < .010

### Associations of SCCs with sociodemographic and clinical variables

Independent samples t-tests and Chi-squared tests were performed to examine differences between patients with and without probable CIs in sociodemographic and clinical variables (see Table 1). None of the sociodemographic variables were shown to be significantly associated with probable CIs. Regarding clinical variables, patients with probable CIs were diagnosed at an older age, *t*(300) = 2.00, *p* = 0.046, *d* = 0.28, were on HD for fewer months, *t*(292) = -2.77, *p* = 0.001, *d* = 0.38, and were more likely to have hypertension, χ^2^(1) = 4.92, p = 0.027, *V* = 0.13, and diabetes, χ^2^(1) = 4.21, p = 0.040, *V* = 0.12, compared to patients with no CI. No other clinical variable was associated with cognitive complaints.

To further explore the role of time on HD, we derived three groups: patients on HD ≤ 6 months (i.e., newly initiated HD); patients between 7–24 months on HD; and patients on HD > 24 months. Rates of probable CIs and continuous SCC scores were compared across the three groups. Results indicated a significantly higher proportion of CIs among the newly initiated patients (32.8%) than the patients on HD for more than 24 months (16.8%), χ^2^(2) = 9.64, p = 0.008, *V* = 0.18. ANOVA comparisons on the continuous KDQOL-CF scores indicated a significant group effect, *F*(2, 294) = 4.61, *p* = 0.011, *η*^*2*^ = 0.03. Post-hoc comparisons showed that newly initiated HD patients (i.e., 0–6 months) had significantly more frequent SCCs (M = 64.88, SD = 23.17) than patients on HD for more than 24 months (M = 73.67, SD = 21.06), *t*(244) = -2.84, *p* = 0.005, *d* = 0.41. Patients on HD for 7–24 months had a mean score that fell between the other two groups but was not significantly different from any of these two groups.

### Associations of SCCs with patient-reported outcomes

#### Group comparisons

Independent samples t-tests were conducted to evaluate differences between the probable CIs group and the no CI group in self-efficacy, self-management skills, treatment adherence, and mood symptoms (see Table 2). Results showed that patients with probable CIs had lower disease self-efficacy, *t*(302) = -5.25, *p* < 0.001, *d* = 0.72, and lower treatment self-efficacy, *t*(302) = -4.96, *p* < 0.001, *d* = 0.68, compared to patients with no CI. Differences were also noted in self-management skills. Patients in the probable CIs group had significantly lower scores than the no CI group in the skills and technique acquisition, *t*(96.06) = -3.38, *p* = 0.001, *d* = 0.52, self-monitoring and insight, *t*(303) = -2.57, *p* = 0.011, *d* = 0.35, and health services navigation domains of the Health Education Impact Questionnaire, *t*(302) = -2.82, *p* = 0.005, *d* = 0.39. With regard to adherence indicators, analyses indicated that patients with probable CIs reported significantly lower medication adherence, *t*(303) = -2.38, *p* = 0.018, *d* = 0.32, and lower adherence in the “times of particular difficulty” subscale of Renal Adherence Behaviour Questionnaire compared to the no CI group, *t*(303) = -2.46, *p* = 0.014, *d* = 0.34. Differences in other Renal Adherence Behaviour Questionnaire subscale scores or total score were not significant. Symptoms of depression [*t*(303) = 6.65, *p* < 0.001, *d* = 0.91] and anxiety [(303) = 7.36, *p* < 0.001, *d* = 1.00] were significantly higher in the probable CIs group than those with no CI.

**Table 2:** Differences between patients with and without probable cognitive impairments in self-reported outcomes

	Total (*N* = 305)	Probable CIs (*N* = 70)	No CI (*N* = 235)	t-test	
	Mean (SD)			*p* value	Cohen's d
Disease self-efficacy	6.06 (1.84)	5.08 (1.92)	6.35 (1.72)	< .001**	0.72
Treatment self-efficacy	7.06 (1.62)	6.25 (1.68)	7.30 (1.52)	< .001**	0.68
Skills and technique acquisition	2.79 (0.41)	2.63 (0.47)	2.84 (0.37)	.001**	0.52
Self-monitoring and insight	3.05 (0.38)	2.95 (0.40)	3.09 (0.37)	.011*	0.35
Health services navigation	2.98 (0.41)	2.86 (0.42)	3.02 (0.40)	.005**	0.39
Medication adherence	3.46 (0.76)	3.27 (0.75)	3.52 (0.76)	.018*	0.33
Renal adherence behaviour questionnaire	91.99 (11.94)	89.75 (11.88)	92.65 (11.91)	.074	0.24
Fluid restrictions	38.53 (6.02)	37.66 (5.65)	38.79 (6.11)	.170	0.19
Potassium/phosphate restrictions	19.94 (2.87)	19.51 (3.19)	20.06 (2.76)	.192	0.19
Self-care	6.90 (2.09)	6.87 (1.98)	6.90 (2.12)	.902	0.02
Times of particular difficulty	19.00 (3.27)	18.16 (3.08)	19.25 (3.30)	.014*	0.34
Sodium restrictions	7.58 (1.78)	7.54 (1.85)	7.59 (1.76)	.861	0.02
Depressive symptoms	8.32 (4.17)	11.04 (3.82)	7.51 (3.92)	< .001**	0.91
Anxious symptoms	7.10 (4.52)	10.31 (4.50)	6.13 (4.07)	< .001**	1.00

*CI* Cognitive impairments, *SD* Standard deviation

* *p* < .050

** *p* < .010

#### Correlation analyses

Correlation tests were conducted between continuous KDQOL-CF scores, self-efficacy, self-management skills, treatment adherence, and mood symptoms (see Table 3). Higher KDQOL-CF scores were associated with higher disease and treatment self-efficacy, better self-management skills, better medication adherence, and lower depressive and anxious symptoms. KDQOL-CF scores were also significantly correlated with the Renal Adherence Behaviour Questionnaire total score and the fluid restrictions, potassium/phosphate restrictions, and times of particular difficulty subscale scores.

**Table 3:** Correlations between patient-reported outcomes

	1	2	3	4	5	6	7	8	8.1	8.2	8.3	8.4	8.5	9
1. KDQOL-CF	-													
2. Disease self-efficacy	0.41**	-												
3. Treatment self-efficacy	0.38**	0.52**	-											
4. Skills and technique acquisition	0.24**	0.47**	0.31**	-										
5. Self-monitoring and insight	0.21**	0.32**	0.32**	0.37**	-									
6. Health services navigation	0.21**	0.33**	0.30**	0.46**	0.46**	-								
7. Medication adherence	0.19**	0.13*	0.33**	0.08	0.13*	0.23**	-							
8. Renal adherence behaviour questionnaire	0.19**	0.21**	0.54**	0.12*	0.29**	0.21**	0.49**	-						
8.1 Fluid restrictions	0.19**	0.20**	0.48**	0.10	0.17**	0.15**	0.45**	0.91**	-					
8.2 Potassium/phosphate restrictions	0.15**	0.07	0.39**	0.03	0.32**	0.15*	0.37**	0.74**	0.53**	-				
8.3 Self-care	0.06	0.21**	0.28**	0.13*	0.18**	0.22**	0.25**	0.40**	0.26**	0.29**	-			
8.4 Times of particular difficulty	0.14*	0.14*	0.34**	0.10	0.17**	0.17**	0.36**	0.73**	0.60**	0.37**	0.01	-		
8.5 Sodium restrictions	0.05	0.09	0.39**	0.07	0.30**	0.11	0.22**	0.63**	0.44**	0.56**	0.17**	0.36**	-	
9. Depressive symptoms	-0.52**	-0.57**	-0.37**	-0.39**	-0.31**	-0.31**	-0.15**	-0.27**	-0.25**	-0.15**	-0.18**	-0.21**	-0.13*	
10. Anxious symptoms	-0.50**	-0.45**	-0.39**	-0.28**	-0.20**	-0.22**	-0.19**	-0.20**	-0.21**	-0.06	-0.08	-0.21**	-0.09	0.73**

*KDQOL-CF* Kidney disease quality of life cognitive function subscale

* *p* < .050

** *p* < .010

#### Mediation analyses

**Fig. 1:**
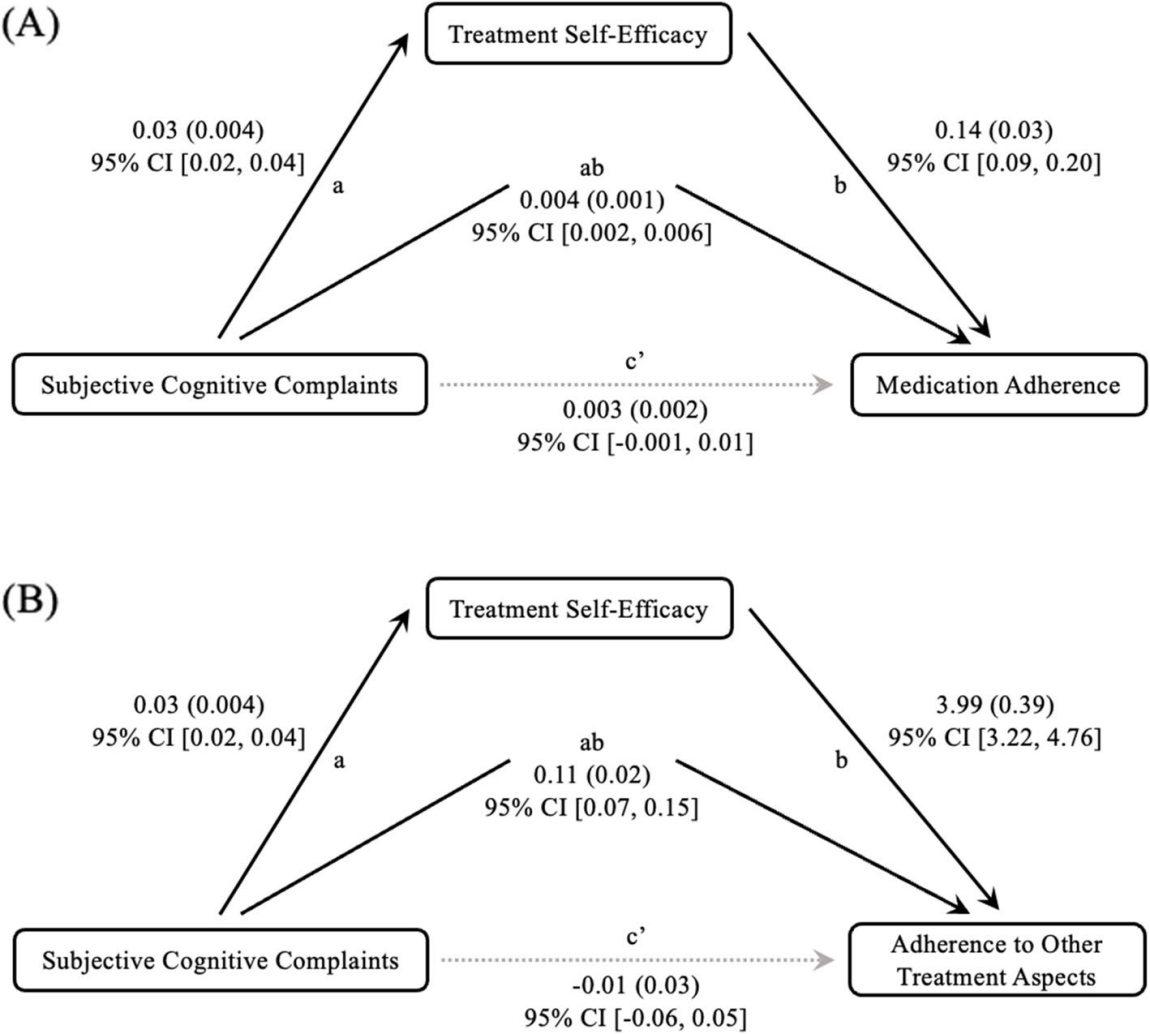
**A** A mediation model where subjective cognitive complaints are indirectly associated with medication adherence through treatment self-efficacy. **B** A mediation model where subjective cognitive complaints are indirectly associated with adherence to other treatment aspects (Renal Adherence Behaviour Questionnaire total score) through treatment self-efficacy. Path a, association between cognitive complaints and self-efficacy. Path b*,* association between self-efficacy and treatment adherence. Path c’, association between cognitive complaints and medication adherence, controlling for the indirect effect. Path ab, the index of the indirect effect. The 95% CI for the indirect paths did not include 0, suggesting that the mediation is significantly different from 0. Solid arrows indicate significant paths. Dashed arrows indicate non-significant paths. Path values represent unstandardised coefficients (standard errors) and 95% CIs

Since the continuous KDQOL-CF scores, treatment self-efficacy, and treatment adherence were all correlated with each other as shown in Table 3, two mediation models were performed to examine the indirect effect of cognitive complaints on medication adherence and on adherence to other treatment aspects (i.e., Renal Adherence Behaviour Questionnaire) through treatment self-efficacy (see Fig. 1). The model with medication adherence as the outcome variable showed a significant indirect effect (standardised indirect effect: *b* = 0.12, *SE* = 0.03, 95% CI [0.06, 0.18]). The model with the Renal Adherence Behaviour Questionnaire total score as the outcome variable also showed a significant indirect effect (standardised indirect effect: *b* = 0.21, *SE* = 0.03, 95% CI [0.14, 0.28]).

## Discussion

The current study found that around 23.0% of HD patients reported frequent cognitive complaints indicative of probable CIs. Patients with probable CIs were diagnosed at an older age and were more likely to have hypertension and diabetes compared to patients with no CI. Patients who newly initiated HD within the past six months were also found to have more frequent SCCs and higher rates of probable CIs compared to patients on HD for more than 24 months. Between-group comparisons and correlational analyses showed that HD patients with more frequent SCCs had lower disease and treatment self-efficacy, poorer self-management skills, poorer treatment adherence, and more severe depressive and anxious symptoms. There was also an indirect effect of SCCs on treatment adherence through treatment self-efficacy.

Nearly a quarter of patients in the current study reported frequent SCCs indicative of clinical CIs, which is similar to a previous study where 24.0% of HD patients reported a KDQOL-CF score below 60 [22]. According to a scoping review, the prevalence rates of objective CIs (as measured by neuropsychological tests) in dialysis patients ranged from 6.6% to 51.0% depending on the neuropsychological tests administered. The observed prevalence of probable CIs in HD patients identified by KDQOL-CF in the current study hence lies within this range but is likely to be an underestimate as prior studies have shown that the cut-off point of 60 on KDQOL-CF, despite its acceptable specificity, has poor sensitivity in detecting CIs [21, 22]. That is, a score higher than 60 may not necessarily indicate intact cognitive function and absence of CI [22]. It is important to note that KDQOL-CF, albeit extensively used in ESRD research, has a limited scope. It comprises only three items (i.e., slow reaction time, concentration difficulty, and confusion) and hence does not assess cognitive difficulties in the critical domains of memory and executive function that have been shown to be most impaired in ESRD patients [5, 7].

Rates of probable CIs were significantly higher among HD patients with diabetes (27.5% in those with diabetes vs. 17.5% in those without) and hypertension (23.8% in those with hypertension and 4.2% in those without), which are known risk factors for both CKD and dementia [10, 52–54]. Vascular disease and cardiovascular risk factors including hypertension and diabetes are thought to be the predominant contributors to ESRD-related CIs. In HD patients, vascular CIs or mixed vascular CIs with Alzheimer’s disease tend to be more common than Alzheimer’s disease alone [10, 54]. The pivotal role of vascular factors in this context is also supported by evidence that cerebrovascular and cardiovascular diseases mainly affect processing speed and executive function, which are the most severely impaired areas in ESRD patients [54, 55]. This is different from Alzheimer’s disease where memory loss is more predominant in its early stages [55]. Future research should determine whether cognitive screening should be prioritised for ESRD patients with these risk factors.

While none of sociodemographic parameters (i.e., age, education, income, employment, etc.) was related to rates of probable CIs, patients diagnosed with ESRD at an older age were more likely to report probable CIs based on the KDQOL-CF. There is a high information load for patients at ESRD diagnosis and dialysis initiation which may be particularly challenging for those diagnosed at older age. These patients may already have age-related cognitive decline or lower cognitive reserve compared to patients who are younger at diagnosis. They may hence become more aware of the cognitive difficulties as they attempt to assimilate, process and act upon the complex medical information related to disease and treatment and as such report more frequent cognitive complaints.

The observed associations between time on dialysis and risk of probable CIs in our sample also suggest that transition onto ERSD and dialysis may potentially intensify any experience of cognitive lapses. Study findings indicated that newly initiated HD patients (i.e., 0–6 months) reported higher rates of probable CIs and more frequent SCCs than patients on HD for more 24 months. It is possible that the initiation of HD may lead to a decrease in cognitive function [55], which contributes to the frequent complaints seen in the first six months after initiation. This is supported by a recent study which found that transition to dialysis was associated with loss of executive function [56]. Also, the novelty of intense physical symptoms related to inter- and intra-dialytic procedures (e.g., fatigue, dizziness) [57–59] may contribute to more frequent SCCs in the incident patients. For new patients these are cognitively taxing symptoms to be managed and regulated. While symptoms are not resolved with longer time on HD, patients over time may learn to adapt and compensate and the symptoms may no longer be as cognitive demanding. The significant difference in SCCs between new incident patients and patients with the longest time on HD is also supported by longitudinal studies that showed improved objective cognitive function [60–62] and decreased SCCs [63] over time on HD treatment. However, within the 179 patients who had been on HD for more than two years in the current study, there were still 30 patients (16.8%) who reported frequent complaints indicative of CIs. Future studies should examine changes in SCCs over time and the risk factors that maintain cognitive complaints in HD patients.

Most notably, study findings indicated significant associations between self-reported cognitive complaints with important patient-reported outcomes, both in terms of skills/capacity as well as actual self-management behaviours. In particular, patients with probable CIs had significantly lower self-efficacy and self-management skills and reported significantly lower adherence (medication intake and adherence in times of particular difficulty) than patients with no CI. These findings were also supported by correlational analyses. ESRD and HD entail complex guidelines related to dietary, fluid, and medication intake that can be further compounded by treatment demands of comorbid conditions. Good disease self-management in ESRD is contingent upon efficient and adequate understanding, processing, and recall of the various information, and translation of this information into appropriate actions and self-management plans [18]. For patients with probable CIs, the cognitive demands of ESRD treatment may be especially onerous, hence undermining capabilities and response efficiency. Our mediation analyses further showed that the effect of SCCs on self-management can be indirect, where the awareness of these cognitive difficulties undermines patients’ self-confidence to perform such cognitively demanding adherence tasks which in turn led to lower adherence. This finding was consistent with Bandura’s social learning theory [64–66]. According to this theory, individuals’ judgements of their capability to perform a certain task (i.e., self-efficacy) is one of the most important regulator of behaviour, and can be influenced by performance accomplishments based on their mastery experiences [65]. If a patient experiences repeated failures in cognitively-demanding self-management activities (e.g., forgetting to take medications), self-confidence to accomplish these tasks in the future will decrease, which in turn contributes to compromised adherence.

To date, the practical implications of CIs in ESRD patients are poorly understood. Few studies have investigated the impact of CIs on self-management behaviour in this population. Hain [67] reported on a sample of 63 HD patients and found that 58.2% of patients with CIs (indicated by Modified Mini-Mental State exam) had evidence of nonadherence based on their attendance at dialysis sessions, serum phosphorus, and interdialytic weight gain. However, no statistical tests were performed to examine the associations between these variables. Two other studies on kidney transplantation recipients found that better everyday problem-solving abilities, assessed by a scenario-based problem-solving task, were associated with better medication adherence [68, 69]. Furthermore, studies in other populations such as community-dwelling older adults [18], heart failure [70, 71], hypertension [72, 73], and type 2 diabetes [74], have all found significant positive associations between cognitive function and treatment adherence. To our knowledge, this is one of the first studies that established the association between cognitive function and self-management in the context of ESRD.

Taken together, these findings have important clinical implications for renal care. First, routine evaluation of SCCs using a simple and quick screening measure (i.e., KDQOL-CF) may help identifying individuals with probable CIs and individuals at risk of low self-efficacy, poor self-management skills, and nonadherence. Currently, CIs in ESRD patients are under-recognised [10, 75]. There is no established protocol or guideline for cognitive screening in the ESRD population. Although neuropsychological tests are considered the gold standard measure of cognitive function, these tests are usually time-consuming, labour-intensive, expensive, and require training of personnel [21, 55]. Brief self-reports may be feasible alternatives in busy clinical settings although their diagnostic ability needs further study. Second, the use of brief screening tool may allow for early intervention for patients at risk of cognitive decline and poorer outcomes. To date, there is limited research on interventions targeting CIs specifically in ESRD patients. Kidney transplantation remains the optimal treatment for ESRD patients and has been shown to improve cognitive function [5]. There are also pharmacological interventions and lifestyle interventions such as exercise and cognitive training that have been shown to improve cognition in other populations such as Alzheimer’s disease, but the majority of these have yet to be tested in ESRD patients [17, 76, 77]. Besides the need for further research on these interventions targeting CIs, it is also important to consider strategies to mitigate/compensate for patients’ everyday cognitive lapses and consequences associated with these lapses. For example, for patients who report adherence difficulties due to memory issues, strategies such as text message reminders, medication management plans, and medication mobile apps may be useful.

Finally, this study replicated the well-established association between SCCs and mood symptoms, which has already been shown in various populations including ESRD [42–45]. Indeed, items measuring mood symptoms sometimes overlap with items measuring SCCs (e.g., difficulty concentrating). Also, individuals with depression and anxiety exhibit cognitive biases which may make them hypervigilant towards negative information such as failure in everyday cognitive tasks, resulting in an overreporting of SCCs [42].

Study limitations warrant acknowledgement. First, the study used a convenience sample and cross-sectional design hence conclusions about directionality of effects, causal inferences, or the longitudinal course of outcomes, cannot be drawn. It is possible that the relationship between SCCs and self-management behaviours is bi-directional and nonlinear. There may also be potential confounding or moderating factors of the association between SCCs and self-management which should be tested in future studies. Although the sample was well representative of the Singapore Renal Registry, it comprised predominantly individuals of Asian ethnicities and of disadvantaged socioeconomic background who volunteered for an observational study or an intervention targeting self-management. Self-selection bias is likely to be present due to the high protocol demands of the studies and possible randomisation into the treatment group. This may limit generalisability of findings and warrant replication in other settings. Second, we excluded patients with documented diagnosis of CIs that may prohibit informed consent and completion of assessments, which means that the prevalence of probable CIs reported in the current study is likely an underestimate. Lastly, since cognitive complaint was not a primary outcome in the original studies, the SCC measure used was limited in its content as it did not assess memory or executive function. Future studies should use more comprehensive SCC measures to identify specific cognitive domains where HD patients may experience cognitive difficulties.

## Conclusions

The current study assessed subjective cognitive complaints in HD patients and examined associations of SCCs with sociodemographic, clinical, and self-management behaviour variables. Results showed that nearly a quarter of patients reported frequent complaints indicative of CIs, and these patients were more likely to be new incident patients, diagnosed at an older age, and have comorbid diabetes and hypertension. More frequent SCCs were also associated with lower disease and treatment self-efficacy, poorer self-management skills, and poorer adherence. In addition, treatment self-efficacy mediated the relationship between SCCs and treatment adherence. Although the SCC measure used in the current study lack sensitivity in detecting objective CIs, findings suggest that cognitive complaints may be a modifiable risk factor of nonadherence in HD patients. Future research is needed to confirm these relationships with more comprehensive SCC measures and longitudinal designs. This line of research will be the foundation of future interventions or support strategies that target cognitive complaints and SCC-related treatment nonadherence in HD patients.

## Data Availability

The datasets used and/or analysed during the current study are available from the corresponding author on reasonable request.

## Abbreviations

ANOVA: Analysis of variance
CCI: Charlson comorbidity index
CI: Cognitive impairment
ESRD: End-stage renal disease
HD: Haemodialysis
IDWGr: Relative interdialytic weight gain
KDQOL-CF: Kidney disease quality of life cognitive function subscale
Kt/V: Dialysis adequacy
M: Mean
NKF: National kidney foundation
SCC: Subjective cognitive complaint
SD: Standard deviation
